# MEASURING COGNITIVE FUNCTION IN OLDER ADULTS WITH SENSORY LOSS

**DOI:** 10.1093/geroni/igad104.0762

**Published:** 2023-12-21

**Authors:** Jennifer Deal, Emma Nichols, Alden Gross, Bonnielin Swenor

**Affiliations:** Johns Hopkins University, Baltimore, Maryland, United States; University of Southern California, Los Angeles, California, United States; Johns Hopkins University Bloomberg School of Public Health, Baltimore, Maryland, United States; Johns Hopkins University, Baltimore, Maryland, United States

## Abstract

Neurocognitive batteries are administered orally or on paper, requiring participants to hear and see to participate; yet 55% of Americans aged 60+ years have hearing or vision loss. We will review 3 studies investigating whether sensory loss could bias cognitive testing. First, we used item response theory to test whether cognitive testing is more difficult (biased) for individuals with vs. without sensory loss, controlling for underlying cognitive function using Atherosclerosis Risk in Communities (ARIC) Study and Baltimore Longitudinal Study on Aging data. Although differential item functioning exists for some tests, it is not salient (>1 standard error difference), suggesting that sensory loss may not have a strong impact on measuring cognitive function in well-designed and conducted research studies. Second, we described how audiometric hearing loss impacts completion of neurocognitive testing, and how missing test scores affect estimates of the hearing loss-cognitive test performance association in a cross-sectional analysis of 3,678 adults (72-94 years) in ARIC. Hearing loss was associated with greater missingness on two auditory-only tests (Logical Memory, Digits Backwards) and two written tests (Boston Naming, Trail Making B). Ignoring this missing data underestimated the hearing-cognition association. Finally, we identified cohort studies measuring cognition in older adults through systematic review, and surveyed investigators for how sensory health was addressed in their studies. We found variation in methods used to assess sensory loss, with implications for resource allocation. Using these studies, we will discuss how to best measure cognition in adults with sensory loss, with a focus on inclusion and equity.

